# Tripartite motif 27 promotes cardiac hypertrophy via PTEN/Akt/mTOR signal pathways

**DOI:** 10.1080/21655979.2022.2051814

**Published:** 2022-03-21

**Authors:** Yan Chen, Zewen Liu, Zhengqing Hu, Xiuyuan Feng, Li Zuo

**Affiliations:** a Department of Cardiology, Institute of Cardiovascular Diseases, First Affiliated Hospital of Wuhan University, Wuhan, Hubei, China; b Department of Cardiology, Ezhou Central Hospital, Wuhan University, Ezhou, China; c Department of Anesthesiology, Ezhou Central Hospital, Wuhan University, Ezhou, China; d Physiology and Biomedical Sciences, Molecular Physiology and Biophysics Laboratory, University of Maine Presque Isle Campus, Presque Isle, ME, USA; e Interdisciplinary Biophysics Program, The Ohio State University, Columbus, OH 43210, USA

**Keywords:** Tripartite motif27, cardiac hypertrophy, signal pathways

## Abstract

Tripartite motif-containing 27 (Trim27) is highly expressed in tumor cells and regulates natural immunity and apoptosis. However, the effects of Trim27 in cardiac hypertrophy are not fully elucidated. In this study, we explored the potential role of Trim27 in pressure overload-induced cardiac hypertrophy and the underlying mechanism. The results indicated that compared to sham operation (Sham) group, transverse aortic constriction (TAC) group showed significantly up-regulated Trim27 protein expression (P < 0.05). The neonatal rat cardiomyocytes (NRCMs) were isolated and stimulated with PBS, angiotensin (AngII) and phenylephrine (PE). NRCMs were collected to detect the protein expression of Trim27. The results were consistent with the results in vivo. Compared to PBS treatment, the expression of Trim27 protein in NRCMs was significantly increased after PE or AngII stimulation (P < 0.05, respectively). Knockout of Trim27 reduced the size of cardiomyocytes and the protein expression of ANP, BNP, and β-MHC, improved cardiac function, and decreased myocardial hypertrophy (P < 0.05). Trim27 may be involved in regulating the development of cardiac hypertrophy. Further results showed that Trim27 enhanced the protein expression of phosphorylation of Akt, GSK3β, mTOR, and P70s6k by interacting with PTEN (phosphatase tensin homolog). These findings revealed that Trim27 can promote cardiac hypertrophy by activating PTEN/Akt/GSK3β/mTOR signaling pathways.

## Introduction

Cardiac hypertrophy (CH) has become an important risk factor that increases the incidence of several cardiovascular diseases (CVDs) [1]. Previous studies reported that pathological CH caused myocardial ischemia, arrhythmia, and even heart failure (HF) [2–4]. Until recently, the underlying mechanisms of pathological CH still remains largely unknown.

The ubiquitin-proteasome system (UPS) is one of the main systems for protein degradation, and it plays a key role in important biological activities [5]. Recently literatures reported that UPS plays important roles in various CVDs including ischemia/reperfusion (I/R) injury, CH, and heart failure [5]. Tripartite motif (TRIM) proteins are a family of UPS that take part in different kinds of cellular processes. TRIM protein, also known as RBCC protein, is the RING component protein of E3 ubiquitin ligase. The structure of the TRIM family contains three conserved domains, and the structure from N-terminal to C-terminal usually contains a RING domain, one or two B-boxes, and a helical coil structure [6]. With the in-depth study of the TRIM protein family, TRIM protein has shown important roles in the physiological and pathological processes. TRIM protein has a variety of expression forms in the cytoplasm and nucleus. It participates in cell growth and differentiation as well as other cell biochemical processes. It also plays a central role in host defense against viral infection and bacterial immune responses [7]. It mainly participates in the interaction between proteins, as an important receptor for selective autophagy [8].

Tripartite motif-containing 27 (Trim27) functions as a key Trim protein family member. Recently, Trim27 was reported to regulate the PI3/Akt signaling pathway, interacting with PTEN, and mediating its poly-ubiquitination [9]. In addition, Trim27 reacts with the IKK kinase family and TBK1 to inhibit activation, negatively regulates NF-κB signaling pathways activated by TNF, IL-1, TLR3, and viral infections, and inhibits IRF3 signaling pathways [10]. Trim27 binds to USP7 to inhibit RIP1ʹs fascine [11], which can positively regulate TNF-α [12]. Trim27-deficient mice can resist hepatocyte apoptosis induced by TNF-α-D galactosamine [13]. Trim27 plays a transcriptional regulatory role by affecting the interaction between the nuclear retinal glioma protein (Rb) and the Mi-2β-containing histone deacetylation complex. In the process of mycobacterial infection, Trim27 promotes both JNK/p38MAPK signaling and apoptosis pathways to limit the innate immune response and apoptosis of macrophages of the phagocytic mycobacteria [14]. At present, the research on Trim27 in disease is not fully explored and more efforts are concentrated on the research of tumor and natural immunity against pathogenic microorganisms [15]. In cancer research, it has been shown that Trim27 can promote tumorigenesis [16]. For example, increased Trim27 expression was seen in colorectal cancer (CRC) tissues [17]. Down-regulating the expression of Trim27 can inhibit the proliferation of ovarian cancer cells both in vivo and in vitro [18]. In addition, Trim27 plays some roles in diseases such as lung cancer, tuberculosis, hepatitis C virus [19], diabetes, Parkinson’s, and allergies [20]. Trim27 also mediated abnormal mesangial cell proliferation in the kidney of lupus [21]. Trim27 was reported to inhibit the kinase activity of PI3KC2β, resulting in reduced KCa3.1 channel activity and a decrease in Ca2+ influx and cytokine production stimulated by T-cell receptor (TCR) [22]. However, the effect of Trim27 on CH remains largely unknown.

Therefore, the present study aimed to determine the role of Trim27 in TAC-induced CH and the potential mechanisms. We hypothesize that Trim27 may be involved in regulating the development of CH.

## Materials and methods

### Animals and reagents

Male wild-type mice (WT) and Trim27 knockout mice (Trim27-KO) were obtained from Animal Experiment Center of Wuhan University, weighing about 24–28 g of 8–10 weeks old, and were selected as experimental subjects to establish a mouse CH model. The mice were divided into four groups (n = 10 in each group): WT Sham group, WT TAC group, KO Sham group, KO TAC group. The Sham group was the sham operation group, and the TAC group was the transverse aortic constriction group. All experimental procedures were conducted according to the guidance of Guidelines for the Care and Use of Laboratory Animals published by the US National Institutes of Health and were approved by the Animal Experiment Center of Wuhan University (Approval Number: L2019-K-06).

### Animal model of pathological CH

**Figure 1.**
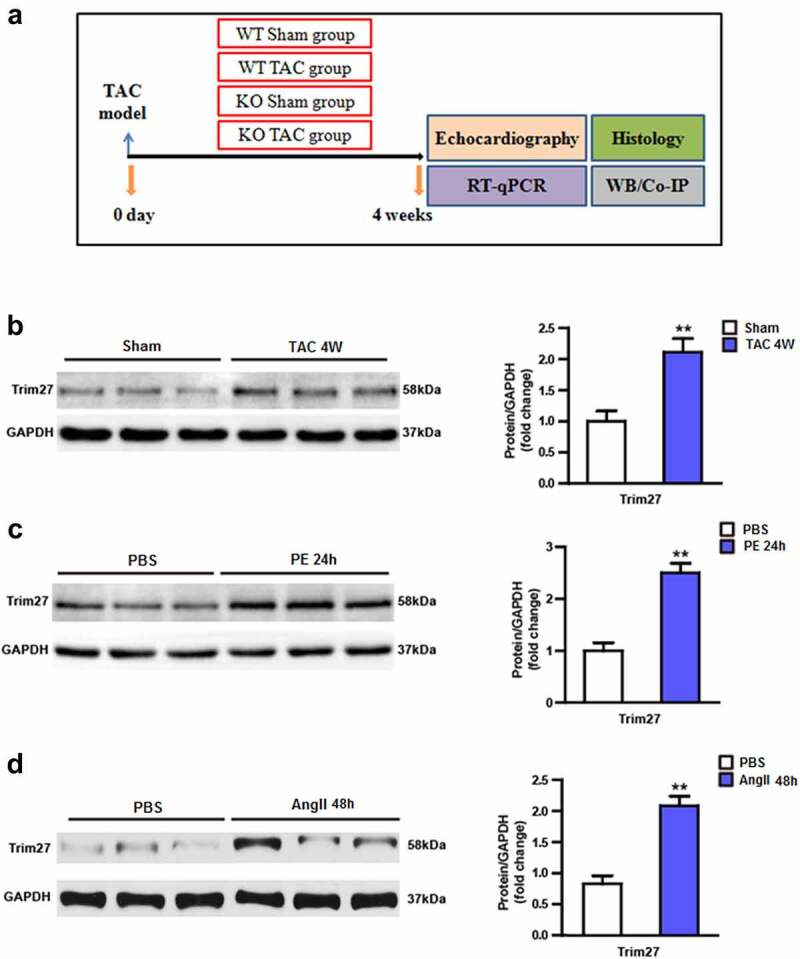
Tripartite motif 27 (Trim27) is induced by hypertrophic stimuli. (a) Experimental protocol of this study. (b) Protein expression of Tripartite motif 27 (Trim27) in transverse aortic constriction (TAC)-induced cardiac hypertrophy. n = 6 mice per group, * P < 0.05 vs. sham. (c) Protein expression of Trim27 in extracts from NRCMs treated with PE or phosphate-buffered solution (PBS). n = 6 per group, (d) Protein expression of Trim27 in extracts from NRCMs treated with AngII or PBS. ** P < 0.01 vs. PBS.

After intraperitoneal anesthesia with 3% sodium pentobarbital (50 mg/kg), the mice were connected to the electrocardiograph (iworx-ECG, US). After the tracheal intubation, a ventilator was used (tidal volume 0.4–0.5 mL; frequency 100 times/min; breathing ratio 1:3). A 7.0 surgical suture was passed through the blood vessel, and a 26 G (25.0–27.5 g mouse) or 27 G (23.5–25.0 g) syringe needle was placed in parallel. The blood vessel and the needle were ligated together, and then the needle was removed to achieve the corresponding degree of blood vessel constriction. After the ligation was completed, sutures were sequentially completed [23]. After the arterial arch ligation, once the mouse has spontaneous breathing and a strong response to the pinch, the tracheal intubation was pulled out, and the mouse was placed in a cage containing autoclaved bedding, feed, and drinking water. The detailed study design was shown in Figure 1a.

### Echocardiography analysis

Echocardiography analysis was performed as previously reported [24]. In brief, echocardiography was conducted under anesthesia with 1.5% – 2% isoflurane, and with a Mylab30CV (ESAOTE) ultrasound system attached to a 15Mz probe. Cardiac measurements included checking of left ventricular end-diastolic diameter (LVEDd), left ventricular end-systolic diameter (LVESd) and short-axis shortening rate (FS).

### Histological analysis

The formalin-embedded hearts were sliced into 6 μm sections that were stained with hematoxylin and eosin (H&E) using wheat germ agglutinin (WGA) to quantify CH and picrosirius red (PSR) to quantify cardiac fibrosis [25].

### Cell culture and treatment

Neonatal rat cardiomyocytes (NRCMs) were prepared as previously described [21]. Briefly, SD rat pups on postnatal days 1–3 were sacrificed by swift decapitation then the hearts were removed and minced immediately. Heart was digested in a solution with 0.08% collagenase type II and 0.125% trypsin and then the supernatant was collected. 0.1 mmol/L Brdu was applied to inhibit cardiac fibroblast proliferation in NRCMs. NRCMs were placed onto 6-well plates and cultured in DMEM/F12 (Gibco, Grand Island, NY, USA) containing 10% FBS (Gibco, Grand Island, NY, USA) for 48 h. Then, the medium was replaced with serum-free DMEM/F12 for 12 h. AdshTrim27 advenoviral was used to knockdown Trim27 in NRCMs and AdTrim27 was used to overexpress Trim27 in NRCMs. AdshRNA, and AdGFP were used for control. These advenoviral were synthesized by GeneChem (Shanghai, China). We investigated the molecular mechanism with phenylephrine (PE, 100uM) for 24 h or angiotensin (AngII, 1uM) for 48 h [26].

### Western blot analysis

Western blotting was conducted to evaluate protein expression levels as described previously. The primary antibody used in this manuscript were: BNP (1:1000, Santa Cruz Biotechnology), β-MHC (1:2000, Sigma), ANP (1:1000, Cell Signaling Technology), p-Akt (1:1000, Cell Signaling Technology), Akt (1:1000, Cell Signaling Technology), p-mTOR (1:1000, Cell Signaling Technology), mTOR (1:1000, Cell Signaling Technology), p-p70S6K (1:1000, Cell Signaling Technology), p70S6K (1:1000, Cell Signaling Technology) and GAPDH (1:1000, Santa Cruz Biotechnology, Santa Cruz, CA, USA). The total protein levels were normalized to GAPDH [27].

### Co-immunoprecipitation (Co-IP)

For IP, whole-cell extracts were prepared after transfection or stimulation with appropriate ligands, followed by incubation overnight with the appropriate antibodies plus Protein A/G beads (Santa Cruz Biotechnology, USA). Beads were washed five times and separated by SDS-PAGE. Western blot was performed by using the antibodies as indicated above [9].

### Statistical analysis

All data are expressed as mean ± SEM. One-way analysis of variance (ANOVA) was carried out to evaluate the differences between the groups in GraphPad Prism software. Differences were regarded as statistically significant at *P* < 0.05.

## Results

In our study, we predicted that Trim27-KO may reverse TAC-induced cardiac hypertrophy. Therefore, we aimed to investigate the role of Trim27 in cardiac hypertrophy and the underlying mechanisms. Our data revealed that Trim27 was significantly increased in response to cardiac hypertrophy both in vivo and in vitro. Moreover, Trim27-KO could alleviate the cardiac hypertrophy and cardiac fibrosis. Trim27 could directly interact with PTEN. Mechanistically, Akt/mTOR/p70s6k may be involved in the protective role of Trim27 against cardiac hypertrophy both in vivo and in vitro. In summary, our findings demonstrated that Trim27 is expected to be a potential target for the therapy of cardiac hypertrophy.

### The expression of Trim27 are increased in response to CH

We firstly examined the expression of Trim27 in CH model both in vivo and in vitro. As shown in Figure 1(b), a TAC group displayed significantly increased Trim27 protein expression compared to sham group (P < 0.05). We then examined the Trim27 protein expression in NRCMs. As exhibited in Figure 1(c), PE-stimulated NRCMs showed markedly elevated Trim27 protein expression compared to PBS-stimulated NRCMs. Moreover, AngII-treated NRCMs also showed increased Trim27 protein expression compared to PBS-stimulated NRCMs (Figure 1(d), p < 0.05). Taken together, the expression of Trim27 were increased in response to CH.

### Trim27-KO reversed the TAC-induced CH in vivo

**Figure 2.**
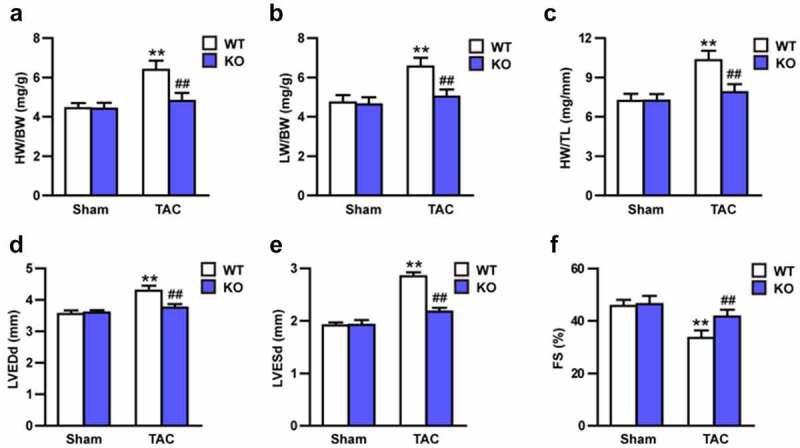
Tripartite motif 27 (Trim27) deficiency in the heart attenuates transverse aortic constriction (TAC)-induced cardiac hypertrophy. (a-c) The ratios of heart weight to body weight (HW/BW), lung weight (LW) /BW, and HW/tibia length (TL) in WT and Trim27- KO mice after sham treatment or TAC for four weeks. n = 8 for each group. (d-f) Left ventricular end-diastolic diameter (LVEDd), left ventricular end-systolic diameter (LVESd), and short-axis shortening rate (FS%) in WT and Trim27- KO mice after sham treatment or TAC. n = 8 for each group. **P < 0.01 vs. WT sham; ## P < 0.01 vs. WT TAC.

**Figure 3.**
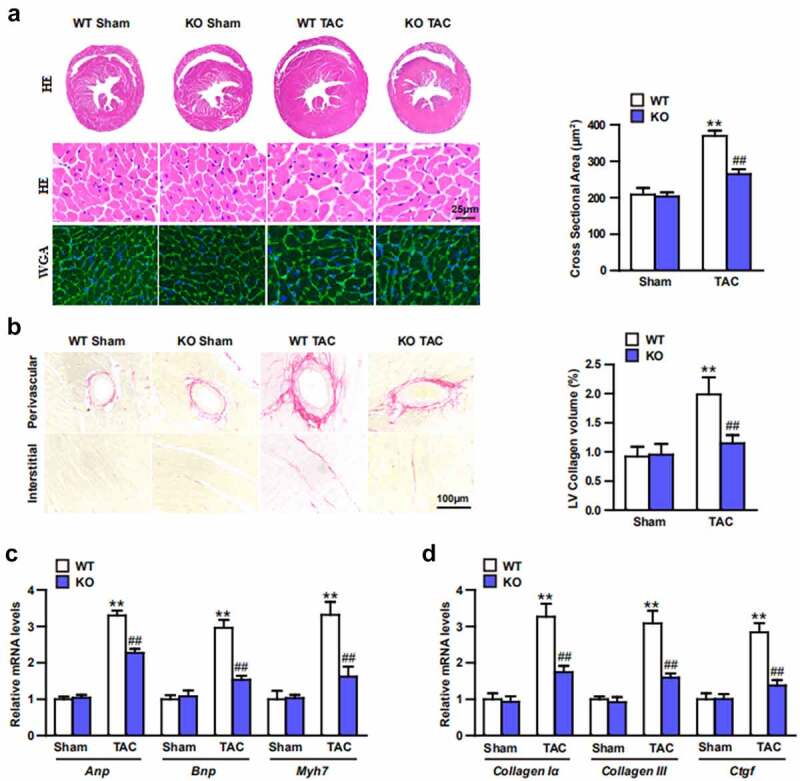
Trim27-Deficient Mice modulates TAC-induced hypertrophy, fibrosis, and heart function in vivo. (a) Sections of hearts from WT and Trim27-KO mice subjected to TAC or Sham treatment were stained with H&E and WGA to analysis heart and cardiomyocyte size and quantification analysis (n = 10 mice per group). (b) Picrosirius red (PSR) staining of histological sections of hearts from sham- and TAC-treated WT or Trim27-KO mice and quantification analysis (n = 10 mice per group). Scale bars, 100 μm. ** P < 0.01 vs. WT sham; ## P < 0.01 vs. WT TAC. (c, d) Hypertrophic markers atrial natriuretic peptide (Anp), B-type natriuretic peptide (Bnp), and β-myosin heavy chain (Myh7) mRNA levels and myocardial fibrosis of the Collagen I, Collagen III and Ctgf in vivo (n = 4 mice per group). ** P < 0.01 vs. WT sham; ## P < 0.01 vs. WT TAC.

Echocardiography were conducted to test the effect of Trim27-KO on cardiac function. 4-weeks after TAC, the TAC mice showed increased LVEDd, LVESd, and decreased FS compared to sham mice (Figure 2(d-f); P < 0.05). Moreover, ratios of HW/BW, LW/BW, and HW/TL were significantly elevated in TAC group compared to sham group. In addition, Trim27-KO markedly decreased these elevated indicators induced by TAC (Figure 2(a-c); P < 0.05). In order to clarify whether Trim27-KO can reduce the CH in vivo, HE staining and WGA staining were utilized to examine the cross-section area of cardiomyocytes. The cross-sectional area of the ventricle in Trim27-KO TAC mice was significantly decreased compared to WT TAC group (Figure 3(a)). In addition, the mRNA expression of ANP, BNP, and β-MHC mRNA in TAC group markedly increased compared to sham group, and the mRNA expression of ANP, BNP, and β-MHC mRNA in Trim27-KO TAC group significantly decreased compared to WT TAC group (Figure 3(c)). It is further verified that the lack of Trim27 inhibits the development of TAC-induced hypertrophy. In the fourth week after receiving TAC treatment, PSR staining showed that increased perivascular and interstitial fibrosis were found in TAC heart compared to sham mice, and Trim27-KO reversed the cardiac fibrosis induced by TAC (Figure 3(b)). We then tested the mRNA expression of collagen I, collagen III, and CTGF, as shown in Figure 3(d), the mRNA expressions of the cardiac fibrotic indicators were markedly increased compared to sham group, and Trim27-KO significantly decreased these cardiac fibrotic indicators induced by TAC. The above clues demonstrated that Trim27 is an endogenous positive regulator of cardiac hypertrophy, and Trim27 can prevent myocardial hypertrophy induced by TAC.

### Knocking down Trim27 inhibited hypertrophy in vitro

**Figure 4.**
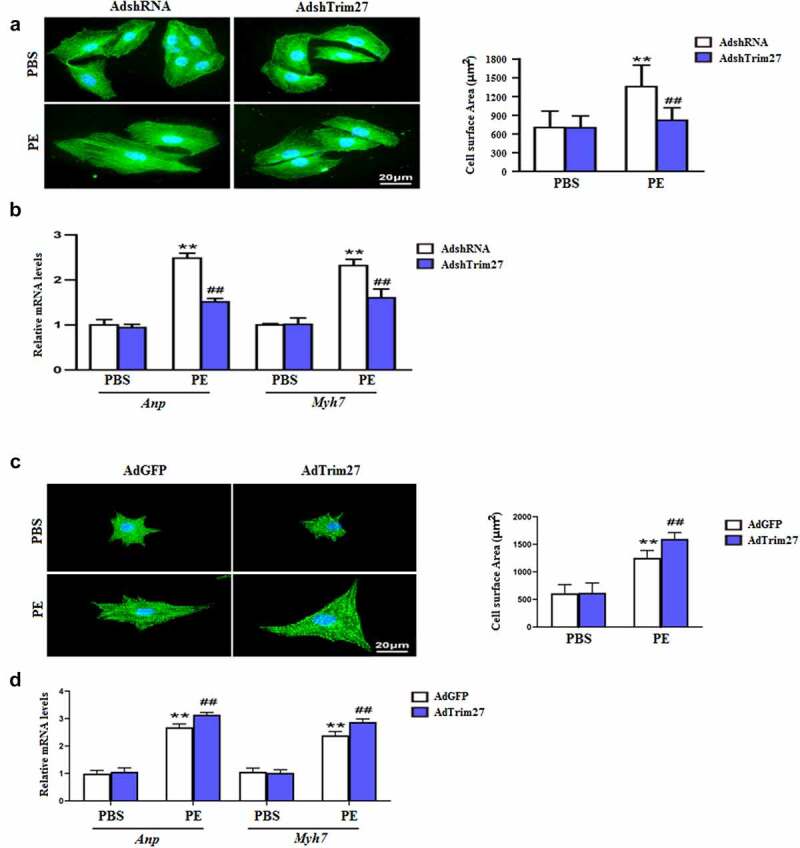
Trim27 promotes phenylephrine (PE)-induced cardiomyocyte hypertrophy. (a) Representative images of cardiomyocytes (immunostained with the α-actinin antibody) infected with Ad-shTrim27 or Ad-shRNA after treatment with PE (100 μM) for 24 hours and quantification analysis (n = 4 independent experiments). Scale bars, 20 μm. **P < 0.01 vs. PBS- AdshRNA, ##P < 0.01 vs. PE- AdshRNA. (b) Atrial natriuretic peptide (ANP) and Myh7 mRNA levels in NRCMs after PE treatment. The real-time polymerase chain reaction analysis was performed. n = 4 per group, **P < 0.01 vs. PBS- AdshRNA, ##P < 0.01 vs. PE- AdshRNA. (c) Representative images of cardiomyocytes (immunostained with the α-actinin antibody) infected with AdTrim27 or AdGFP after treatment with PE (100 μM) for 24 hours and quantification analysis (n = 4 independent experiments). Scale bars, 20 μm. **P < 0.01 vs. PBS-AdGFP, ##P < 0.01 vs. PE-AdGFP. (d) Atrial natriuretic peptide (ANP) and Myh7 mRNA levels in NRCMs after PE treatment. The real-time PCR analysis was performed. n = 4 per group, **P < 0.01 vs. PBS-AdGFP, ##P < 0.01 vs. PE-AdGFP.

We then examined the effect of Trim27 knockdown on hypertrophy in vitro. As exhibited in Figure 4(a), PE-stimulated NRCMs showed an increase in cardiomyocyte surface area (CSA) compared to PBS-stimulated NRCMs, and NRCMs treated with AdshTrim27 displayed a significant reduction in CSA (P < 0.05). Furthermore, we tested the mRNA expressions of cardiac hypertrophy markers, ANP and Myh7 in vitro. Increased mRNA expression of ANP and Myh7 were found in PE-stimulated NRCMs compared to PBS-stimulated NRCMs, and knocking down Trim27 with AdshTrim27 markedly decreased the increased mRNA expression of ANP and Myh7 induced by PE-stimulated. Next, we evaluated whether overexpressed Trim27 have effect on hypertrophy in vitro. As exhibited in Figure 4(c), overexpressed Trim27 with AdTrim27 in PE-stimulated NRCMs significantly increased CSA compared to PE-stimulated NRCMs. Moreover, AdTrim27 plus PE-treated NRCMs showed markedly increased mRNA expression of ANP and Myh7 compared to AdGFP or PE-treated NRCMs (Figure 4(d), p < 0.05).

### Akt/mTOR signaling contribute to the protective role of Trim27 against CH

**Figure 5.**
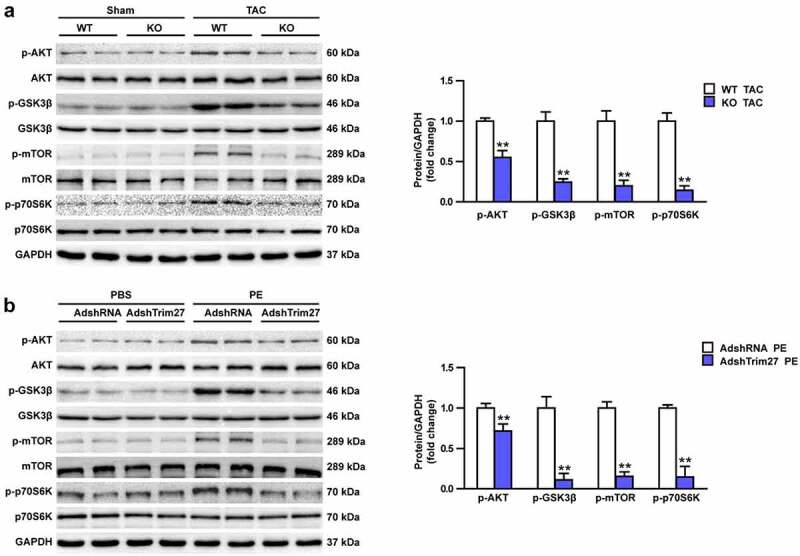
Effects of Trim27 on the phosphorylation levels of Akt, GSK3β, mTOR, and p70s6k. (a) Representative Western blots of the phosphorylated and the total level of Akt, GSK3β, mTOR, and p70s6k in WT cardiomyocytes hypertrophy treated by TAC and Trim27-KO group. Quantification of the relative changes in phosphorylation of Akt, GSK3β, mTOR, and p-p70s6k (n = 4 mice per group). ** P < 0.01 vs. WT-TAC. (b) Representative Western blots of the phosphorylated and the total level of Akt, GSK3β, mTOR, and p70s6k in neonatal rat cardiomyocytes with PBS-and PE-induced cardiomyocytes. Quantification of the relative changes in phosphorylation of Akt, GSK3β, mTOR, and p-p70s6k. GAPDH was used as a loading control. ** P < 0.01 vs. AdshRNA/PE (n = 4 per group).

Akt/GSK3β/mTOR/p70s6k signaling pathway were widely reported to play vital roles in CH. However, whether Akt/GSK3β/mTOR/p70s6k signaling cascade is involved in the protective role of Trim27 in response to CH remains unclear. We then firstly tested the protein expressions of phosphorylated-Akt, GSK3β, mTOR, and p70s6k in vivo. As shown in Figure 5(a), markedly increased protein expressions of the above phosphorylated protein expression were observed in WT TAC group compared to WT sham group (P < 0.05). Trim27-KO notably inhibit Akt/GSK3β/mTOR/p70s6k signaling pathway, which indicated by decreased protein expressions of p-Akt, GSK3β, mTOR, and p70s6k in Trim27-KO TAC mice compared to WT TAC mice (P < 0.05, Figure 5(a)). Then, we tested the protein expressions of p-Akt, p-GSK3β, p-mTOR, and p-p70s6k in PE-induced NRCMs. As exhibited in Figure 5(b), the expressions of p-Akt, p-GSK3β, p-mTOR, and p-p70s6k were significantly increased in PE-stimulated NRCMs compared to PBS-stimulated NRCMs and the expressions of Akt, GSKβ, mTOR, and p70S6K were declined in AdshTrim27 group compare to Adsh-RNA group (P < 0.05) (Figure 5(b)). Altogether, these results indicated that Akt/GSK3β/mTOR/p70s6k signaling contribute to the protective role of Trim27 against CH.

### Trim 27 interacted with PTEN in NRCMs

**Figure 6.**
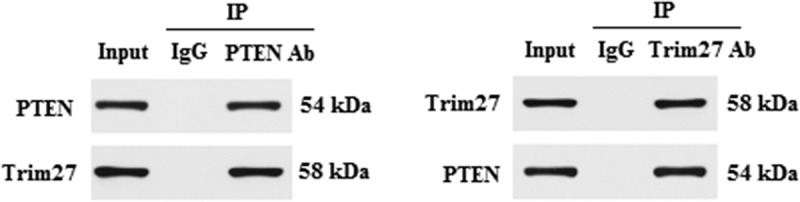
Trim 27 interacted with PTEN in NRCMs. Co-IP assay was performed to examine the interaction between Trim27 and PTEN in NRCMs.

PTEN is identified as a certified critical component in Akt pathway. PTEN/Akt cascade was reported to be involved in regulating CH. In order to further test the function of Trim27 in Akt signaling, we analyzed the interaction between Trim27 and PTEN by co-immunoprecipitation (Co-IP). As shown in Figure 6, it was easier to identify that there was a stronger interaction between Trim27 and PTEN, indicated that Trim27 directly interacted with PTEN in NRCMs.

## Discussion

The present study mechanistically tests the effect of Trim27 on CH and clarifies for the first time that Trim27 may possibly serve as a potential cardio-protective factor against CH both in vitro and in vivo. The precise mechanism involved in the protective role of Trim27 maybe interacted with PTEN to inhibit the activation of Akt/GSK3β/mTOR/p70s6k signaling pathways.

CH is the most critical pathophysiological condition involved in response to stress, which may include hypertrophy, fibrosis, and systolic dysfunction. Under persistent pressure-overload condition, the compensatory CH may transform to decompensated CH, leading to arrhythmia or heart failure ultimately [28]. The complex pathophysiological mechanism of CH has always been the focus of multiple scientific studies [29], and thus, the precise mechanisms of CH are remained to be further explored.

Trim proteins were reported in regulating many different cellular functions and biological processes, including cell proliferation, cancer transformation, autophagocytosis, post-translational modifications, etc [30]. Trim27 belongs to the Trim protein family, which was reported to take part in several kinds of diseases including colitis [17], lupus nephritis [21], diabetes [31] and ovarian cancer [16]. Recently, Trim27 was reported to involved in myocardial ischemia/reperfusion (MI/R) by interacting with p53 [32]. Whereas the protective effect of Trim27 on CH still need to be clarified. Our data demonstrated that Trim27-KO treatment effectively inhibited TAC-induced cell surface area and improved TAC-induced cardiac changes. We also found that Trim27-KO significantly improved cardiac function, inhibited TAC-induced CH parameters (HW/BW, LW/BW, and HW/TL) changes. In addition, CH marker mRNA expression (ANP, BNP, and β-MHC) are also reduced. These results initially demonstrated that Trim27 can protect against TAC-induced CH. Furthermore, we also found that knocking down Trim27 by AdshTrim27 significantly attenuated the PE-induced hypertrophy, and overexpress Trim27 with AdTrim27 aggravated PE-induced hypertrophy. Therefore, these results indicated that Trim27 can protect against CH both in vivo and in vitro. The precise mechanism of Trim27 against TAC-induced CH is largely unknown.

Vascular smooth muscle cells (VSMCs) constantly express contractile phenotypic markers, which are decreased markedly in response to injury. The biological role of Trim27 was investigated in VSMCs. Trim27 cooperatively maintains the endogenous expression of PDGFR beta and contractile phenotype of human VSMCs [33,34]. The Akt signaling pathway plays a pivotal role in cell proliferation and cardiac protective mechanisms-ischemic preconditioning and ischemic postconditioning [35]. The E3 ubiquitin ligase, Trim27 prohibits FcepsilonRI activation of KCa3.1 and downstream signaling by ubiquitinating and impeding PI3KC2beta. The class II phosphatidylinositol-3-kinase C2beta (PI3KC2beta) is necessary for Fcepsilon RI-stimulated activation of KCa3.1, and degranulation of bone marrow-derived mast cells (BMMC). These findings identify Trim27 as a negative regulator of mast cells in vivo. Furthermore, previous studies demonstrated that the mTOR signaling was involved in CH process [36]. In the present study, we found that Trim27 could increase excessive phosphorylation of the total level of Akt, GSK3β, mTOR, and p70s6k both in vivo and in vitro per response to CH. And knocking down Trim27 with AdshTrim27 could notably inhibit the Akt/GSK3β/mTOR/p70s6k pathway. Therefore, we assumed that Trim27 increased excessive hypertrophy activity via Akt signaling pathways in response to CH.

However, how Trim27 regulates Akt/GSK3β/mTOR/p70s6k signaling pathways in the present study remains unclear. Numerous studies demonstrated that PTEN/Akt signaling pathway contributed to the pathological process of CH [37,38]. In addition, previous study reported that Trim27 could directly interact with PTEN to modulate apoptosis of esophageal squamous cell carcinoma (ESCC) and accelerate its glucose uptake by regulating PI3K/Akt signaling pathways [9]. Hence, we also evaluated whether Trim27 could interact with PTEN in response to CH. Using Co-IP analysis we found that Trim27 could directly interact with PTEN in response to CH in the present study.

Taken together, the present study provided evidence that Trim27 may modulate pathological CH via interacting with PTEN to regulate Akt/GSK3β/mTOR/p70s6k signaling pathways. Thus, these results further verify the notion that Trim27 can be a potential therapeutic target for preventing CH. It also indicates the possibility of inhibiting the Trim27 activation as an anti-CH drug during clinical application.

## Conclusion

gIn general, our work illustrated that Trim27 could promote CH by activating Akt/GSK3β/mTOR signaling via directly interacted with PTEN. Trim27-KO could reverse CH via inhibiting Akt/GSK3β/mTOR/p70s6k signaling pathway. Trim27 is expected to a potential target of CH in the future.

## Supplementary Material

Supplemental Material

## References

[1] Ba L, Gao J, Chen Y, et al. Allicin attenuates pathological cardiac hypertrophy by inhibiting autophagy via activation of PI3K/Akt/mTOR and MAPK/ERK/mTOR signaling pathways. Phytomedicine. 2019;58:152765. 31005720 10.1016/j.phymed.2018.11.025

[2] Kohli S, Ahuja S, Rani V. Transcription factors in heart: promising therapeutic targets in cardiac hypertrophy. Curr Cardiol Rev. 2011;7(4):262–271. 22758628 10.2174/157340311799960618 PMC3322445

[3] Shang L, Pin L, Zhu S, et al. Plantamajoside attenuates isoproterenol-induced cardiac hypertrophy associated with the HDAC2 and AKT/ GSK-3beta signaling pathway. Chem Biol Interact. 2019;307:21–28. 31009642 10.1016/j.cbi.2019.04.024

[4] Teekakirikul P, Zhu W, Huang HC, et al. Hypertrophic cardiomyopathy: an overview of genetics and management. Biomolecules. 2019;9(12):878. 10.3390/biom9120878 PMC6995589 31888115

[5] Gupta I, Varshney NK, Khan S. Emergence of members of TRAF and DUB of ubiquitin proteasome system in the regulation of hypertrophic cardiomyopathy. Front Genet. 2018;9:336. 30186311 10.3389/fgene.2018.00336 PMC6110912

[6] Mohammad Mahabub-Uz Zaman TN, Takagi T, Okamura T, et al. Ubiquitination-Deubiquitination by the TRIM27-USP7 complex regulates tumor necrosis factor alpha-induced apoptosis. Mol Cell Biol. 2013;33(24):4971–4984. 24144979 10.1128/MCB.00465-13 PMC3889550

[7] van Gent M, Sparrer KMJ, Gack MU. TRIM proteins and their roles in antiviral host defenses. Annu Rev Virol. 2018;5(1):385–405. 29949725 10.1146/annurev-virology-092917-043323 PMC6186430

[8] Hatakeyama S. TRIM family proteins: roles in autophagy, immunity, and carcinogenesis. Trends Biochem Sci. 2017;42(4):297–311. 28118948 10.1016/j.tibs.2017.01.002

[9] Ma L, Yao N, Chen P, et al. TRIM27 promotes the development of esophagus cancer via regulating PTEN/AKT signaling pathway. Cancer Cell Int. 2019;19(1):283. 31719796 10.1186/s12935-019-0998-4 PMC6839104

[10] Zheng Q, Hou J, Zhou Y, et al. Siglec1 suppresses antiviral innate immune response by inducing TBK1 degradation via the ubiquitin ligase TRIM27. Cell Res. 2015;25(10):1121–1136. 26358190 10.1038/cr.2015.108 PMC4650625

[11] Cai J, Chen HY, Peng SJ, et al. USP7-TRIM27 axis negatively modulates antiviral type I IFN signaling. FASEB J. 2018;32(10):5238–5249. 29688809 10.1096/fj.201700473RR

[12] Zaman MM, Shinagawa T, Ishii S. Trim27 -deficient mice are susceptible to streptozotocin-induced diabetes. FEBS Open Bio. 2013b;4(1):60–64. 10.1016/j.fob.2013.12.002 PMC3879403 24392305

[13] Zaman MM, Nomura T, Takagi T, et al. Ubiquitination-deubiquitination by the TRIM27-USP7 complex regulates tumor necrosis factor alpha-induced apoptosis. Mol Cell Biol. 2013a;33(24):4971–4984. 24144979 10.1128/MCB.00465-13 PMC3889550

[14] Wang J, Teng JL, Zhao D, et al. The ubiquitin ligase TRIM27 functions as a host restriction factor antagonized by Mycobacterium tuberculosis PtpA during mycobacterial infection. Sci Rep. 2016;6(1):34827. 27698396 10.1038/srep34827 PMC5048167

[15] Conwell SE, White AE, Harper JW, et al. Identification of TRIM27 as a novel degradation target of herpes simplex virus 1 ICP0. J Virol. 2015;89(1):220–229. 25320289 10.1128/JVI.02635-14 PMC4301158

[16] Ma Y, Wei Z, Bast RC Jr., et al. Downregulation of TRIM27 expression inhibits the proliferation of ovarian cancer cells in vitro and in vivo. Lab Invest. 2016;96(1):37–48. 26568293 10.1038/labinvest.2015.132

[17] Zhang HX, Xu ZS, Lin H, et al. TRIM27 mediates STAT3 activation at retromer-positive structures to promote colitis and colitis-associated carcinogenesis. Nat Commun. 2018;9(1):3441. 30143645 10.1038/s41467-018-05796-z PMC6109048

[18] Jiang J, Xie C, Liu Y, et al. Up-regulation of miR-383-5p suppresses proliferation and enhances chemosensitivity in ovarian cancer cells by targeting TRIM27. Biomed Pharmacother. 2019;109:595–601. 30399596 10.1016/j.biopha.2018.10.148

[19] Zheng F, Xu N, Zhang Y. TRIM27 promotes hepatitis C virus replication by suppressing Type I interferon response. Inflammation. 2019;42(4):1317–1325. 30847745 10.1007/s10753-019-00992-5

[20] Srivastava S, Cai X, Li Z, et al. Phosphatidylinositol-3-kinase C2beta and TRIM27 function to positively and negatively regulate IgE receptor activation of mast cells. Mol Cell Biol. 2012;32(15):3132–3139. 22645315 10.1128/MCB.00019-12 PMC3434511

[21] Liu J, Feng X, Tian Y, et al. Knockdown of TRIM27 expression suppresses the dysfunction of mesangial cells in lupus nephritis by FoxO1 pathway. J Cell Physiol. 2019;234(7):11555–11566. 30648253 10.1002/jcp.27810

[22] Cai X, Srivastava S, Sun Y, et al. Tripartite motif containing protein 27 negatively regulates CD4 T cells by ubiquitinating and inhibiting the class II PI3K-C2beta. Proc Natl Acad Sci U S A. 2011;108(50):20072–20077. 22128329 10.1073/pnas.1111233109 PMC3250182

[23] Pan J, Xu Z, Guo G, et al. 2021Circ_nuclear factor I X (circNfix) attenuates pressure overload-induced cardiac hypertrophy via regulating miR-145-5p/ATF3 axis. Bioengineered. 2021;12(1):5373–5385. 34468254 10.1080/21655979.2021.1960462 PMC8806771

[24] Shuai W, Kong B, Fu H, et al. Loss of MD1 increases vulnerability to ventricular arrhythmia in diet-induced obesity mice via enhanced activation of the TLR4/MyD88/CaMKII signaling pathway. Nutr Metab Cardiovasc Dis. 2019;29(9):991–998. 31353205 10.1016/j.numecd.2019.06.004

[25] Li Z, Guo Z, Lan R, Cai S, Lin Z, Li J, Wang J, Li Z, Liu P. The poly(ADP-ribosyl)ation of BRD4 mediated by PARP1 promoted pathological cardiac hypertrophy. Acta Pharm Sin B. 2021;11(5):1286–1299. 34094834 10.1016/j.apsb.2020.12.012 PMC8148063

[26] Li Y, Shi Y, He Y, et al. RNA binding Motif protein-38 regulates myocardial hypertrophy in LXR-α-dependent lipogenesis pathway. Bioengineered. 2021b;12(2):9655–9667. 34854353 10.1080/21655979.2021.1977552 PMC8809983

[27] Ciocci Pardo A, Diaz RG, Gonzalez Arbelaez LF, et al. Benzolamide perpetuates acidic conditions during reperfusion and reduces myocardial ischemia-reperfusion injury. J Appl Physiol. 2018;125(2):340–352. 1985. 29357509 10.1152/japplphysiol.00957.2017

[28] Rababa’h AM, Guillory AN, Mustafa R, et al. Oxidative stress and cardiac remodeling: an updated edge. Curr Cardiol Rev. 2018;14(1):53–59. 29332590 10.2174/1573403X14666180111145207 PMC5872263

[29] Lampada A, Hochhauser D, Salomoni P. Autophagy and receptor tyrosine kinase signalling: a mTORC2 matter. Cell Cycle. 2017b;16(20):1855–1856. 28934018 10.1080/15384101.2017.1372548 PMC5638352

[30] Jaworska AM, Wlodarczyk NA, Mackiewicz A, et al. The role of TRIM family proteins in the regulation of cancer stem cell self-renewal. Stem Cells. 2020;38(2):165–173. 31664748 10.1002/stem.3109 PMC7027504

[31] Zaman MM, Shinagawa T, Ishii S. Trim27 -deficient mice are susceptible to streptozotocin-induced diabetes. FEBS Open Bio. 2013;4(1):60–64. 10.1016/j.fob.2013.12.002 PMC3879403 24392305

[32] Li Y, Meng Q, Wang L, et al. TRIM27 protects against cardiac ischemia-reperfusion injury by suppression of apoptosis and inflammation via negatively regulating p53. Biochem Biophys Res Commun. 2021a;557:127–134. 33865220 10.1016/j.bbrc.2021.03.061

[33] He B, Tang RH, Weisleder N, et al. Enhancing muscle membrane repair by gene delivery of MG53 ameliorates muscular dystrophy and heart failure in delta-Sarcoglycan-deficient hamsters. Mol Ther. 2012;20(4):727–735. 22314291 10.1038/mt.2012.5 PMC3321592

[34] Wang Y, Hao Y, Zhao Y, et al. TRIM28 and TRIM27 are required for expressions of PDGFRbeta and contractile phenotypic genes by vascular smooth muscle cells. FASEB J. 2020;34(5):6271–6283. 32162409 10.1096/fj.201902828RR

[35] Braz JC, Gill RM, Corbly AK, et al. Selective activation of PI3Kalpha/Akt/GSK-3beta signalling and cardiac compensatory hypertrophy during recovery from heart failure. Eur J Heart Fail. 2009;11(8):739–748. 19633101 10.1093/eurjhf/hfp094

[36] Yu P, Zhang Y, Li C, et al. Class III PI 3K-mediated prolonged activation of autophagy plays a critical role in the transition of cardiac hypertrophy to heart failure. J Cell Mol Med. 2015;19(7):1710–1719. 25851780 10.1111/jcmm.12547 PMC4511367

[37] Tian M, Jiang X, Li X, et al. LKB1IP promotes pathological cardiac hypertrophy by targeting PTEN/Akt signalling pathway. J Cell Mol Med. 2021;25(5):2517–2529. 33486894 10.1111/jcmm.16199 PMC7933949

[38] Yang H, Wang XX, Zhou CY, et al. Tripartite motif 10 regulates cardiac hypertrophy by targeting the PTEN/AKT pathway. J Cell Mol Med. 2020;24(11):6233–6241. 32343488 10.1111/jcmm.15257 PMC7294125

