# Supplementary figures and images for: Tripartite motif 27 promotes cardiac hypertrophy via PTEN/Akt/mTOR signal pathways
Source: Bioengineered. 2022 Mar 21;13(4):8321–31. doi: 10.1080/21655979.2022.2051814 (PMC9208448; doi:10.1080/21655979.2022.2051814)

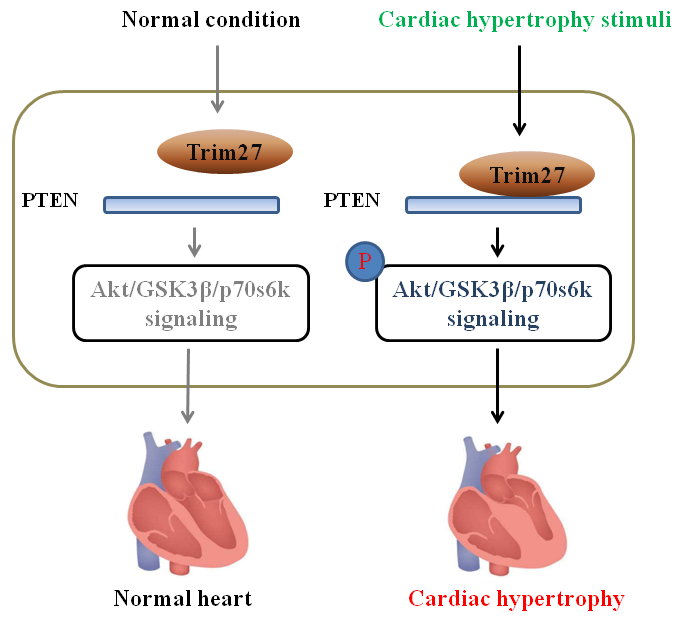

Supplement: Supplemental Material [file KBIE_A_2051814_SM5533.tif]
